# The Landscape of Aminoacyl-tRNA Synthetases Involved in Severe Acute Respiratory Syndrome Coronavirus 2 Infection

**DOI:** 10.3389/fphys.2021.818297

**Published:** 2022-01-26

**Authors:** Yajuan Feng, Kang Tang, Qi Lai, Jingxian Liang, Min Feng, Zhong-Wei Zhou, Haissi Cui, Xiangjun Du, Han Zhang, Litao Sun

**Affiliations:** ^1^School of Public Health (Shenzhen), Shenzhen Campus of Sun Yat-sen University, Shenzhen, China; ^2^Institute of Medical Biology, Chinese Academy of Medical Sciences and Peking Union Medical College, Kunming, China; ^3^School of Medicine, Shenzhen Campus of Sun Yat-sen University, Shenzhen, China; ^4^Department of Molecular Medicine, The Scripps Research Institute, La Jolla, CA, United States

**Keywords:** aminoacyl-tRNA synthetase, coronavirus, SARS-CoV-2, interaction network, the shortest path

## Abstract

Aminoacyl-tRNA synthetases (aaRSs) are essential enzymes in translation by linking amino acids onto their cognate tRNAs during protein synthesis. During evolution, aaRSs develop numerous non-canonical functions that expand the roles of aaRSs in eukaryotic organisms. Although aaRSs have been implicated in viral infection, the function of aaRSs during infections with coronaviruses (CoVs) remains unclear. Here, we analyzed the data from transcriptomic and proteomic database on human cytoplasmic (cyto) and mitochondrial (mt) aaRSs across infections with three highly pathogenic human CoVs, with a particular focus on severe acute respiratory syndrome coronavirus 2 (SARS-CoV-2). We found an overall downregulation of *aaRSs* at mRNA levels, while the protein levels of some mt-aaRSs and the phosphorylation of certain aaRSs were increased in response to SARS-CoV-2 infection. Strikingly, interaction network between SARS-CoV-2 and human aaRSs displayed a strong involvement of mt-aaRSs. Further co-immunoprecipitation (co-IP) experiments confirmed the physical interaction between SARS-CoV-2 M protein and TARS2. In addition, we identified the intermediate nodes and potential pathways involved in SARS-CoV-2 infection. This study provides an unbiased, overarching perspective on the correlation between aaRSs and SARS-CoV-2. More importantly, this work identifies TARS2, HARS2, and EARS2 as potential key factors involved in COVID-19.

## Introduction

Aminoacyl-tRNA synthetases (aaRSs) is an ancient enzyme family that links specific amino acids onto their cognate tRNAs. In human, cytoplasmic (cyto) and mitochondrial (mt) protein synthesis are mediated by distinct aaRSs, where the cyto- and mt-aaRSs are encoded by the *aaRS1* and *aaRS2* genes, respectively. In higher eukaryotes, some cyto-aaRSs remain free-standing in the cytoplasm while others are components of the multi-synthetase complex (MSC). The mammalian MSC contains nine cyto-aaRSs (EARS1, PARS1, IARS1, LARS1, MARS1, QARS1, KARS1, RARS1, and DARS1), and three non-enzymatic aaRS-interacting multi-functional proteins (AIMP), including AIMP1 (p43), AIMP2 (p38), and AIMP3 (p18). Among them, EARS1 and PARS1 are linked together to form EPRS1.

Despite well-documented function in protein synthesis, aaRSs, AIMPs and the components of MSC have recently been characterized as cellular sensors or immunoregulators in viral infections. For example, WARS1, encoded by the *WARS1* gene, elicits an innate immune response as a cytokine and functions as a cellular entry factor for enteroviruses (Yeung et al., 2018). AIMP1 is secreted to stimulate an anti-viral immune response (Liang et al., 2017). EPRS1 can be released from the MSC and is involved in the anti-viral response of transmissible gastroenteritis coronavirus (TGEV), a coronavirus (CoV) that causes a life-threatening infection in pigs (Galan et al., 2009; Marquez-Jurado et al., 2015). Given a close link between aaRSs and viruses, we preliminarily generated an overall interaction network between human aaRSs and viruses based on public database (Supplementary Figure 1 and Supplementary Table 1). Indeed, we found a strong involvement of aaRSs during viral infection process. These viruses include human immunodeficiency virus 1 (HIV-1), influenza A virus, Zika virus, Ebola virus, etc. However, the systematical analysis of functional connection between human aaRSs and coronaviruses (CoVs), especially the current pandemic of severe acute respiratory syndrome coronavirus 2 (SARS-CoV-2) infection, is still missing.

In humans, SARS-CoV-2 infection leads to a serious multisystem disease named coronavirus disease 2019 (COVID-19), which is characterized with symptoms ranging from asymptomatic infections to acute respiratory distress, causing over five million deaths worldwide since its outbreak (Wiersinga et al., 2020; Wu F. et al., 2020; Asselah et al., 2021; Perico et al., 2021). The ongoing COVID-19 pandemic retains its impact on global health, and the novel SARS-CoV-2 variants spread easier and quicker, increasing the severity of the pandemic. Aside from SARS-CoV-2 infection, infections with other CoVs took place in the past, such as SARS-CoV-1 in 2002 (Stadler et al., 2003; Christian et al., 2004; Wang et al., 2006) and Middle East respiratory syndrome coronavirus (MERS-CoV) in 2012 (Zumla et al., 2015; de Wit et al., 2016), which are all highly pathogenic human CoVs. Unfortunately, since the pathogenesis of CoVs infection as well as the virus-host interaction remain incompletely understood, no specific and efficient therapies are available to date.

To address the pathogenesis of CoVs infections, omics studies on either viral genomes or the host cells have been conducted in substantial numbers. These data have revealed multiple molecular and cellular changes in both clinical samples and infected cells. By mining these publicly available data, we investigated the expression profiling of human aaRSs across infections with three highly pathogenic human CoVs, and analyzed the interaction networks between CoV-proteins and human aaRSs. Intriguingly, the results of mt-aaRSs interacting predominantly with CoV-proteins surprised us. Further experiments confirmed the physical interaction between SARS-CoV-2 M protein and TARS2. In addition, we identified the intermediate nodes that may regulate downstream aaRSs upon SARS-CoV-2 infection, and uncovered the potential signaling pathways involved in this process. Our study provided insights as to which aaRSs represent response key factors or promising targets for pan-coronavirus infections and, especially for SARS-CoV-2 infection.

## Materials and Methods

### Data Sources

Bioinformatic data used in this study include: transcriptomics data from bronchoalveolar lavage fluid (BALF) (Xiong et al., 2020) and post-mortem lung samples (Blanco-Melo et al., 2020) of patients with COVID-19; RNA sequencing (RNA-seq) data of A549-ACE2 cells (Blanco-Melo et al., 2020) and human bronchial epithelial (NHBE) cells (Vishnubalaji et al., 2020) infected with SARS-CoV-2; proteomics data of BALF (Zeng et al., 2021), peripheral blood mononuclear cells (PBMCs) (Li et al., 2021), and liver cells (Leng et al., 2021) from COVID-19 patients, as well as from SARS-CoV-2-infected human Caco-2 cells (Klann et al., 2020); genome-wide CRISPR screening of Vero-E6 cells following three CoVs infections (Wei et al., 2021); interaction data between aaRSs and three CoVs in Vero-E6 cells (Gordon et al., 2020), and the phosphorylation data in Vero-E6 cells infected with SARS-CoV-2 (Bouhaddou et al., 2020). The log2 fold changes (log2 FC) of aaRSs are derived from each dataset.

### *Z*-Scores From Genome-Wide Clustered Regularly Interspaced Short Palindromic Repeats Screening Data

To assess the roles of aaRSs upon infections with three CoVs, the *Z* scores of aaRSs were downloaded, where a positive *Z* score (*Z* > 0) indicates a gene that is pro-viral and confers resistance to virus-induced cell death, while a negative *Z* score (*Z* < 0) indicates a gene that is anti-viral and sensitizes a cell to virus-induced cell death (Wei et al., 2021).

### Analysis of Translation- and Ribosome-Related Genes and Proteins

The list of translation- and ribosome-related genes and proteins was determined based on Gene Ontology (GO) terms (Ashburner et al., 2000) including “cytoplasmic translation” (GO:0002181, *n* = 86), “mitochondrial translation” (GO:0032543, *n* = 37), “cytosolic ribosome” (GO:0022626, *n* = 76), and “mitochondrial ribosome” (GO:0005761, *n* = 24). Gene symbols are converted by the org.Hs.eg.db package in R and their expressions are displayed in heatmap using the ComplexHeatmap package in R.

### Establishment of Interaction Networks

Pathogen-host interactions (PHI) including HPIDB (Ammari et al., 2016) and VirHostNet (Guirimand et al., 2015), as well as BioGRID (Oughtred et al., 2019), DIP (Salwinski et al., 2004), IntAct (Orchard et al., 2014), and MINT (Licata et al., 2012) were utilized for analysis. Gene symbols for aaRSs were downloaded from the Ensembl database using an ID mapping table. After removing the redundant and genetic interactions, 104 unique aaRS-virus interactions were obtained from 30 human aaRSs and 30 viruses.

To analyze the network between aaRSs and CoV-proteins, virus-host protein-protein interactions (PPIs) between aaRSs and SARS-CoV-2, SARS-CoV-1 and MERS-CoV were collected (Gordon et al., 2020) for analysis, respectively.

### Plasmid Construction

The genome of SARS-CoV-2 was a kindly gift from Professor Ji-An Pan (Jin et al., 2021). The full-length cDNA encoding human TARS2 (NP_001258824.1), HARS2 (NP_001265660.1) or NARS2 (NP_001230180.1) was cloned into a pcDNA 3.0 vector, which carries a 3× FLAG tag. SARS-CoV-2 M protein was cloned into a pcDNA 6.0 vector, which carries a 6× HIS-tag. The primers used for cloning are shown in Supplementary Table 2, where F1/R1 was used for amplifying the target gene while F2/R2 was used for amplifying the vector. All clones were constructed using Seamless Clone Construction with KOD-Plus DNA polymerases (TOYOBO, Shanghai, China). Plasmids were validated by Sanger sequencing (TSINGKE biotech, Guangzhou, China), and were purified using an EndoFree Mini Plasmid Kit II (Tiangen, Beijing, China) in accordance with the manufacturer’s instructions.

### Cell Culture and Transfection

HEK293T cells were maintained in DMEM (Gibco, Shanghai, China) supplemented with 10% FBS (ExCell Bio, Shanghai, China) in a humidified atmosphere containing 5% CO_2_ at 37°C. Plasmids were co-transfected in HEK293T cells using Lipofectamine 2000 (Invitrogen, Carlsbad, CA, United States) according to the manufacturer’s instructions. Cells were harvested 48 h post-transfection.

### Co-immunoprecipitation

Cells were washed by ice-cold PBS and collected in Lysis Buffer (50 mM Tris–HCL pH 7.4, 150 mM NaCl, 1 mM EDTA, and 1 mM PMSF, supplemented with a complete EDTA-free protease inhibitor tablet and phosphatase inhibitor cocktail). Lysates were then incubated with Protein G or A agarose beads (Invitrogen, Carlsbad, CA, United States) for binding overnight at 4°C. Beads were washed with Wash Buffer (50 mM Tris–HCL pH 7.4, 150 mM NaCl, 1 mM EDTA, and 1 mM PMSF, supplemented with a complete EDTA-free protease inhibitor tablet and phosphatase inhibitor cocktail) for three times. Proteins were eluted in 1.5× SDS sample buffer for subsequent immunoblotting.

### Western Blot and Antibodies

Proteins were separated on 12% SDS-PAGE gels and then transferred onto a 0.45 μm polyvinylidene fluoride (PVDF) membranes in transfer buffer. The membranes were blocked with 5% non-fat milk in PBS with Tween 20 for 1 h at 37°C and were incubated with specific antibodies overnight at 4°C. The antibodies used for immunoblots include: anti-FLAG M2 (Sigma-Aldrich, St Louis, MO, United States), anti-HIS (Proteintech, Rosemont, IL, United States), and anti-Tubulin (Ray antibody, Beijing, China). Immunoblotting was visualized using a Bio-Rad ChemiDoc system (Bio-Rad, Shanghai, China).

### Identification of Intermediate Genes and Potential Pathways Following Severe Acute Respiratory Syndrome Coronavirus 2 Infection

To identify the relevant genes that may regulate downstream aaRSs upon SARS-CoV-2 infection, the differential aaRSs were divided into the downregulated mt-aaRSs (Group A), the downregulated cyto-aaRSs (Group B), and the upregulated mt-aaRSs (Group C). The shortest paths between the physical interacting (PI) proteins with SARS-CoV-2 and each group of aaRSs proteins were computed, respectively. The intermediate (IN) genes (nodes) for each group were obtained based on a filter of physical PPIs (Pavel et al., 2021). The Benjamin-Hochberg method was used to calculate adjusted *p*-values, where a gene with an adjusted *p*-value (p.adjust) less than 0.05 was considered as a significant IN node. Significant IN node set in each group was further tested for GO terms (Harris et al., 2004) and kyoto encyclopedia of genes and genomes (KEGG) pathway enrichment analysis (Kanehisa et al., 2017) using the clusterProfiler R package (Yu et al., 2012).

### Statistical Analysis

*P* values were converted from the Z scores by using the function pnorm in R, and *p < 0.05 and **p < 0.01 were considered statistically significant.

## Results

### The Potential Roles of Aminoacyl-tRNA Synthetases Upon Infections With Three Highly Pathogenic Coronaviruses

To determine the potential roles of aaRSs in the pathogenic commonalities among infections with CoVs, we firstly analyzed a transcriptional profile from a genome-wide CRISPR screening database (Wei et al., 2021) in Vero-E6 cells challenged with SARS-CoV-2, wild type MERS-CoV (WT, EMC/2012), tissue culture-adapted MERS-CoV-T1015N, and the bat-CoV HKU5 expressing SARS-CoV-1 spike protein (HKU5-SARS-CoV-1-S), respectively. The replication-competent vesicular stomatitis virus (VSV) expressing SARS-CoV-2 spike protein (rVSV-SARS-CoV-2-S) was used as a surrogate for viral entry of SARS-CoV-2. The *Z* scores were derived directly from the original screening, where a positive *Z* score (*Z* > 0) suggests a gene may have a pro-viral function while a negative *Z* score (*Z* < 0) suggests an anti-viral role (Wei et al., 2021).

**FIGURE 1:**
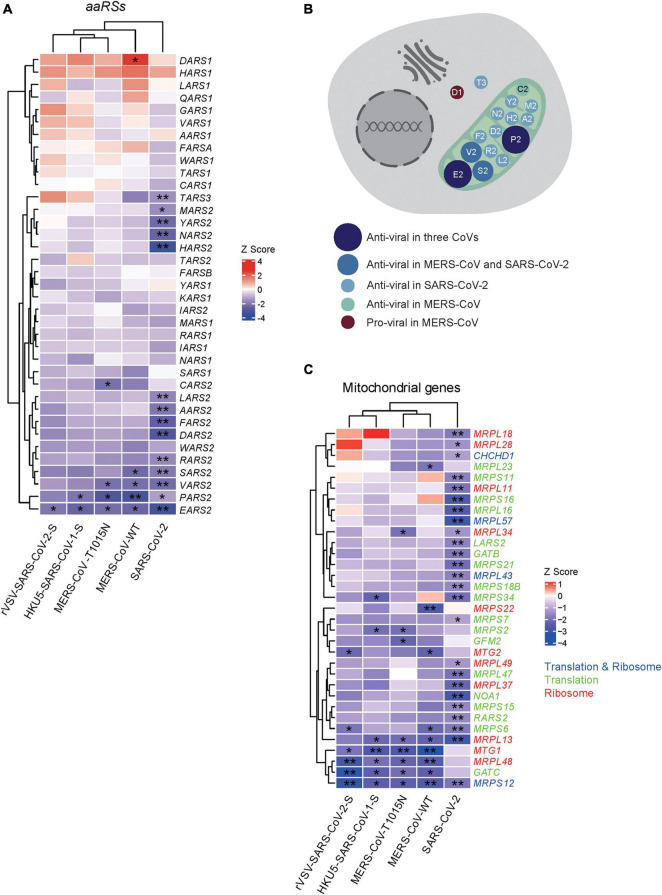
Genome-wide CRISPR-based analysis of human aminoacyl-tRNA synthetases (aaRSs) upon infections with three coronaviruses (CoVs). **(A)** *Z* scores of *aaRSs* in Vero-E6 cells challenged with three CoVs, including SARS-CoV-2, MERS-CoV (WT), MERS-CoV-T1015N, HKU5-SARS-CoV-1-S, and rVSV-SARS-CoV-2-S, respectively. Transcriptional expressions of *aaRSs* based on a genome-wide CRISPR screening were clustered by their mean *Z* scores with red representing *Z* > 0 while blue representing *Z* < 0. *p < 0.05; **p < 0.01 (vs. mock-infected cells). **(B)** Subcellular localization of aaRSs upon CoVs infections based on their *Z* scores, where the mitochondrion is shown in light green. **(C)** Z scores of mitochondrial translation- and ribosome-related genes in Vero-E6 cells challenged with different CoVs. Transcriptional expressions of translation- and ribosome-related genes (blue), translation-related genes (green), and ribosome-related genes (red) based on a genome-wide CRISPR screening were clustered by their mean *Z* scores with red representing *Z* > 0 while blue representing *Z* < 0. *p < 0.05; **p < 0.01 (vs. mock-infected cells).

As shown in Figure 1A, 14 *aaRSs* (*MARS2*, *TARS3*, *LARS2*, *AARS2*, *RARS2*, *SARS2*, *PARS2*, *EARS2*, *HARS2*, *DARS2*, *FARS2*, *NARS2*, *YARS2*, and *VARS2*) (mean *Z* = −2.14), 2 *aaRSs* (*PARS2* and *EARS2*) (mean *Z* = −2.21), and 4 *aaRSs* (*VARS2, SARS2, PARS2*, and *EARS2*) (mean *Z* = −2.44) showed significant negative *Z* scores upon infections of SARS-CoV-2, HKU5-SARS-CoV-1-S, and MERS-CoV, respectively, suggesting that these *aaRSs* play putative anti-viral roles during viral infection. By contrast, *DARS1*, with a significant positive *Z* score (*Z* = 2.43), exhibited a likely pro-viral role upon MERS-CoV infection.

Next, we compared the *Z* scores of human *aaRSs* genes across CoVs infections. *PARS2* and *EARS2* showed significant negative *Z* scores upon infections with SARS-CoV-2, HKU5-SARS-CoV-1-S, and MERS-CoV (WT and tissue-adapted), suggesting possible roles as pan-coronavirus inhibitors. In addition, *EARS2* displayed a significant negative *Z* score upon infection with rVSV-SARS-CoV-2-S, implying a role in SARS-CoV-2 entry process (Figure 1A). Notably, the changes of aaRSs in most samples were insignificant. The reason might be due to the importance of aaRSs in translation and protein synthesis. Furthermore, the discrepancies in the number of affected aaRSs among different viruses may reflect the distinct pathogenesis across different CoVs.

Intriguingly, further analysis of the *Z* scores in terms of their subcellular localization indicated that the majority of aaRSs with significant *Z* scores come from mitochondria, and these mt-aaRSs are likely to play anti-viral roles (Figure 1B), suggesting a key function of mt-aaRSs during CoVs infections. Similarly, a consistent trend in the changes of translation- and ribosome-related genes was observed, both as a whole (Supplementary Figure 2) and in the mitochondria (Figure 1C).

### Transcriptional Profile of *Aminoacyl-tRNA Synthetases* in Patient-Derived Samples and Cell Lines Infected With Severe Acute Respiratory Syndrome Coronavirus 2

**FIGURE 2:**
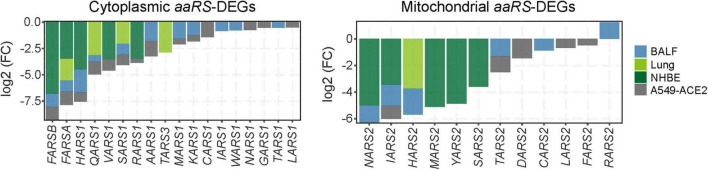
Differentially expressed *aminoacyl-tRNA synthetases (aaRSs)* in patient-derived samples and cell lines infected with SARS-CoV-2. Transcription profile of *aaRS*-DEGs (with the cyto-aaRSs on the left and the mt-aaRSs on the right) in BALF, post-mortem lung tissues from coronavirus disease 2019 (COVID-19) patients, as well as in NHBE and A549-ACE2 cells infected with SARS-CoV-2.

Given the immediacy of the current SARS-CoV-2 pandemic, we next focused on the roles of aaRSs upon SARS-CoV-2 infection. To analyze the effect of SARS-CoV-2 infection on transcription of *aaRSs*, gene expression omnibus (GEO) datasets were utilized (Clough and Barrett, 2016), and the differentially expressed *aaRS* genes (*aaRS*-DEGs) were analyzed. Compared to healthy individuals, most cyto- and mt-aaRSs were downregulated in clinical samples from BALF and post-mortem lung tissues of patients with COVID-19, and the same holds true in SARS-CoV-2-infected human bronchial epithelial (NHBE) cells and A549-ACE2 cells compared to control cells (Figure 2 and Supplementary Table 3). This finding suggested that SARS-CoV-2 infection leads to an overall downregulation of *aaRSs* in patient-derived samples and infected cell lines. Consistently, the translation- and ribosome-related DEGs in both cytoplasm and mitochondria were also downregulated in lung tissues and SARS-CoV-2-infected cell lines; however, their mRNA levels in BALF sample were upregulated, especially in the cytoplasm (Supplementary Figure 3), reflecting the differences between BALF and other samples. On the other hand, this finding implied that the downregulation of cyto-aaRSs in BALF from COVID-19 patients may exert non-canonical functions.

### Protein Profile and Phosphorylated Modification of Aminoacyl-tRNA Synthetases Upon Severe Acute Respiratory Syndrome Coronavirus 2 Infection

**FIGURE 3:**
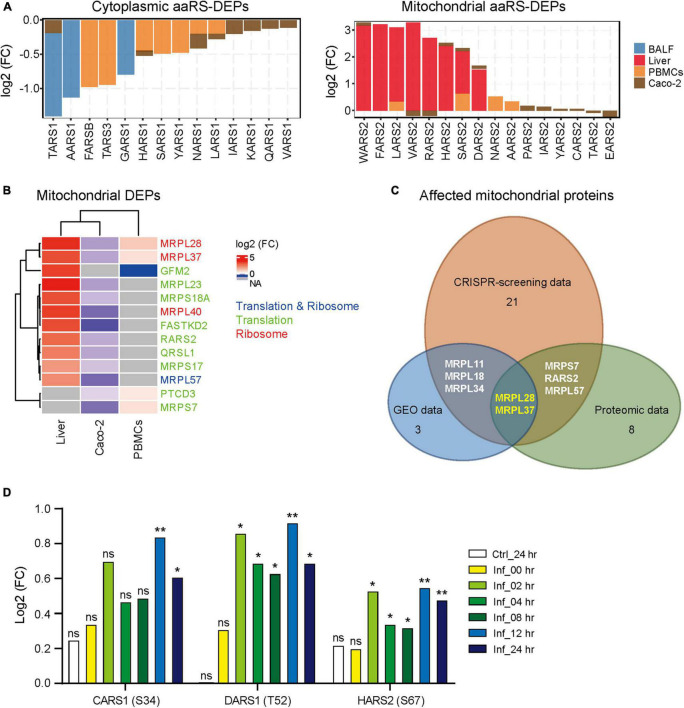
Protein profile and phosphorylated modification of aminoacyl-tRNA synthetases (aaRSs) upon SARS-CoV-2 infection. **(A)** Protein levels of aaRS-DEPs (with the cyto-aaRSs on the left and the mt-aaRSs on the right) in BALF, liver cells and PBMCs from coronavirus disease 2019 (COVID-19) patients, as well as in Caco-2 cells infected with SARS-CoV-2. **(B)** Heatmap of mitochondrial translation- and ribosome-related DEPs from different samples. The fold change in protein expression is indicated by color intensity, with red representing the upregulated DEPs and blue representing the downregulated DEPs. The DEPs include translation- and ribosome-related proteins (blue), translation-related proteins (green), and ribosome-related proteins (red). **(C)** Wayne diagram of the affected mitochondrial translation- and ribosome-related genes and proteins across CRISPR-screening, gene expression omnibus (GEO), and proteomic data. Genes with significant *Z* scores upon SARS-CoV-2 infection, mitochondrial DEGs and DEPs were included for analysis. The remaining number of the affected mitochondrial proteins in each platform is indicated except for the overlapping ones. **(D)** Phosphorylation of aaRSs (phosphorylated site) at different indicated time. Ctrl, control; Inf, infection; hr, hour; ns, not significant; *p < 0.05; **p < 0.01 (vs. mock-infected cells at 0 h).

We next analyzed the protein levels of aaRSs upon SARS-CoV-2 infection across different protein profiles (Klann et al., 2020; Leng et al., 2021; Li et al., 2021; Zeng et al., 2021). The differentially expressed aaRS proteins (aaRS-DEPs) compared to healthy controls were analyzed. Unexpectedly, we found distinct changes of cyto- and mt-aaRS proteins after SARS-CoV-2 infection: most of cyto-aaRSs were downregulated, consistent with their unchanged or downregulated mRNA levels, whereas some mt-aaRSs were remarkably increased in abundance, especially in liver cells from COVID-19 patients (Figure 3A and Supplementary Table 3). The question arises on why mRNA levels of mt-aaRSs are decreased whereas their protein abundance increases. The possible explanations might include the differential regulation of expression, stability and degradation of mRNAs as compared with those of proteins. It is supported by a study demonstrating that the mitochondrial proteins can be stabilized in response to rapamycin treatment, leading to uncorrelated changes in mRNA and protein levels (Fournier et al., 2010). Therefore, the result suggested a post-transcriptionally or post-translationally regulation of a certain set of mt-aaRSs upon SARS-CoV-2 infection, although the molecular mechanisms underlying this finding need further investigation.

Next, we analyzed the translation- and ribosome-related DEPs derived from the same proteomic data. As shown in Supplementary Figure 4A, most of translation and ribosome proteins in cytoplasm were downregulated, the same trend as the changes of cyto aaRS-DEPs. By contrast, the mitochondrial translation and ribosome proteins in liver cells from COVID-19 patients were dramatically increased after SARS-CoV-2 infection, in accordance with the changes of mitochondrial aaRS-DEPs as well (Figure 3B). Given a better correlation between mt-aaRSs and mitochondrial translation- and ribosome-related proteins across CRISPR-screening, GEO and proteomic platforms, we further analyzed the affected mitochondrial proteins. We found 2 affected mitochondrial ribosome proteins, MRPL28 and MRPL37, overlapping three platforms (Figure 3C), suggesting a potential association between mt-aaRSs and mitochondrial proteins upon SARS-CoV-2 infection.

Interestingly, analysis from another proteomic dataset in Vero-E6 cells (Bouhaddou et al., 2020) revealed that the phosphorylation of CARS1 at S34 was significantly increased at 12 and 24 h post-SARS-CoV-2 infection; the phosphorylation of DARS1 at T52 and the phosphorylation of HARS2 at S67 were increased at 2, 4, 8, 12, and 24 h post-infection compared to control cells (Figure 3D). In addition, there were no significant changes of their total proteins after SARS-CoV-2 infection compared to control cells (Supplementary Figure 4B), indicating that phosphorylation mediated post-translation modification of specific aaRSs may play important roles in response to SARS-CoV-2, although no studies of these modifications have been characterized so far.

### Interaction Networks of Coronaviruse-Proteins With Human Aminoacyl-tRNA Synthetases

**FIGURE 4:**
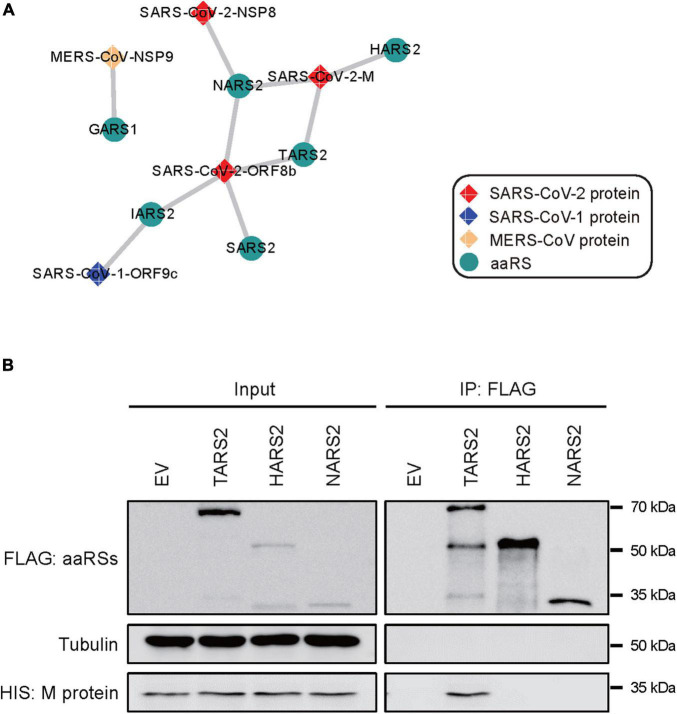
Interaction networks between CoV-proteins and human aminoacyl-tRNA synthetases (aaRSs). **(A)** Physical interactions of CoV-proteins with human aaRSs. Viral proteins are shown as diamonds and the interacting aaRSs are shown as circles. **(B)** Interaction of SARS-CoV-2 M protein with TARS2, HARS2, and NARS2 in human HEK293T cells using co-immunoprecipitation (co-IP) assays. An empty vector (EV) was used as a negative control.

To establish the functional interaction between CoV-proteins and human aaRSs, we mined the proteomics from the ProteomeXchange database to figure out whether CoV-proteins interact with aaRSs. Indeed, we found interactions of SARS-CoV-2 M protein (main protein) with TARS2, NARS2, and HARS2; SARS-CoV-2 ORF8b protein with NARS2, TARS2, IARS2, and SARS2; SARS-CoV-2 NSP8 protein (non-structural protein 8) with NARS2; SARS-CoV-1 ORF9c protein with IARS2, as well as MERS-CoV NSP9 (non-structural protein 9) with GARS1 (Figure 4A), suggesting a complex interaction network between human aaRSs and CoV-proteins.

To validate the robustness of this interaction network, we performed experimental confirmation using co-immunoprecipitation (co-IP) targeting the interaction of SARS-CoV-2 M protein with TARS2, HARS2, and NARS2, respectively. In human HEK293T cells, we verified the physical interaction between SARS-CoV-2 M protein and TARS2, but we did not find the interaction among the others (Figure 4B).

### Identification of the Intermediate Genes Following Severe Acute Respiratory Syndrome Coronavirus 2 Infection

Next, we want to clarify the connections of SARS-CoV-2 proteins with human aaRSs. Proteins that interact with SARS-CoV-2 can be considered as the first responders to the virus, functioning upstream in the host response to the viral infection, while the differential aaRSs derived from the proteomics data represent late effectors in the host immune response (Pavel et al., 2021). To find the gap that links the upstream responders with the downstream effectors, we then identified the intermediate (IN) genes between the two events. The shortest path analysis is an approach to identify the interactor nodes between them. We thus computed the shortest paths between the upstream SARS-CoV-2-interacting proteins and the downstream differential aaRSs proteins to get a novel set of IN genes (nodes). Based on our proteomic analyses (Figure 3A), the aaRS-DEPs were divided into three groups: the downregulated mt-aaRSs (Group A), the downregulated cyto-aaRSs (Group B), and the upregulated mt-aaRSs (Group C) (Supplementary Table 4).

We ultimately got 102, 61, and 453 significant IN nodes for Group A, B, and C, respectively (Supplementary Table 5). Among them, we found 16 IN genes overlapping three groups (Table 1), in which there were four *aaRSs* including *EARS2*, *IARS1*, *IARS2*, and *TARS1*, suggesting that these aaRSs may serve as important mediators linking the upstream SARS-CoV-2 infection and the downstream differential aaRSs. In addition, *EGFR*, a cell surface protein that binds to epidermal growth factor, was also revealed as an overlapped IN gene across three groups (Table 1). Notably, EGFR has been identified as a component of the COVID-19-induced cytokine storm (Kong et al., 2020), and serves as a host factor in promoting viral entry, survival and propagation for SARS-CoV-2 (Klann et al., 2020; Xu et al., 2021). Therefore, this finding implied the notion that its downstream aaRSs proteins may play pivotal roles in COVID-19 mediated by EGFR.

**TABLE 1:** Significant intermediate (IN) genes overlapping three groups.

No.	IN genes	Gene ID
1	*CCT5*	22948
2	*CCT6A*	908
3	*CDH2*	1000
4	*CEP135*	9662
5	*CYB5R3*	1727
6	*EARS2*	124454
7	*ECSIT*	51295
8	*EGFR*	1956
9	*IARS1*	3376
10	*IARS2*	55699
11	*JUP*	3728
12	*RPS14*	6208
13	*SDCBP*	6386
14	*TARS1*	6897
15	*UBA1*	7317
16	*UBA52*	7311

### Gene Ontology and KEGG Enrichment Analyses of the Intermediate Nodes

**FIGURE 5:**
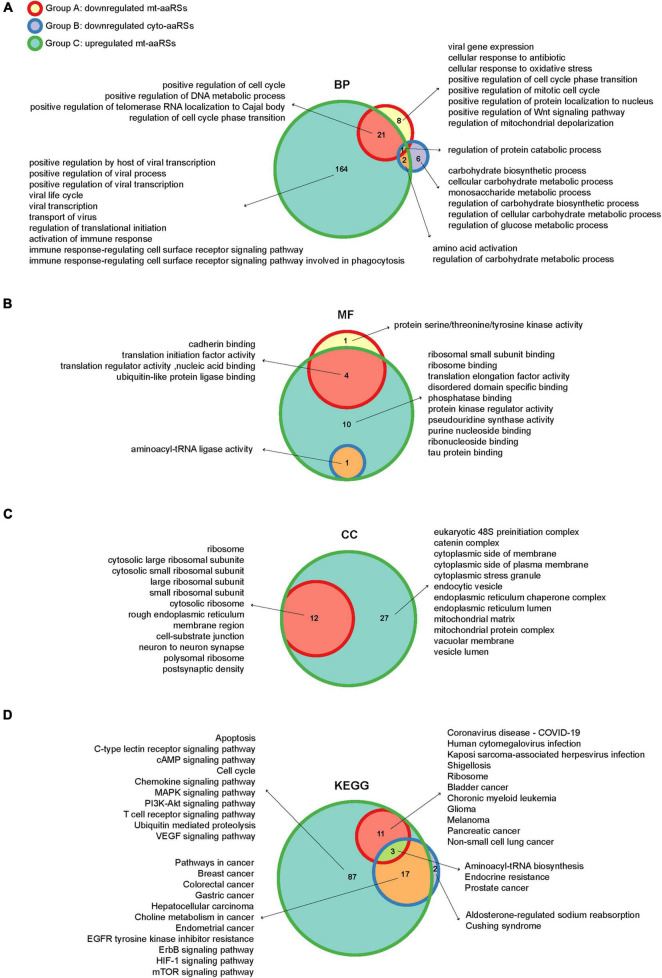
Enriched gene ontology (GO) terms and KEGG pathways of the intermediate (IN) nodes. Wayne diagrams of enriched GO terms in biological process **(A)**, molecular function **(B)** and cellular component **(C)** categories, as well as enriched KEGG pathways **(D)** for IN nodes in different groups. BP, biological process; MF, molecular function; CC, cellular component.

To further depict the functions of the downstream aaRSs, we performed GO and KEGG analyses of the IN nodes in each group (Supplementary Table 5). In biological process (BP) category of GO terms, we got one enriched term “regulation of protein catabolic process” overlapping three groups (Figure 5A). In particular, the IN nodes from Group C were mostly enriched in “positive regulation of viral process,” “viral life cycle,” “viral transcription,” and “activation of immune response”; by contrast, IN nodes from Groups A and B were mostly enriched in cellular regulation and responses including cell cycle, biosynthetic and metabolic processes (Figure 5A). Notably, the enriched GO terms in molecular function (MF) and cellular component (CC) categories for the significant IN nodes from all groups were generally canonical roles of aaRSs, such as “aminoacyl-tRNA ligase activity,” “translation initiation factor activity,” “ribosome,” and “mitochondrial protein complex” (Figures 5B,C). Collectively, these findings suggested that while most aaRSs play canonical roles, the upregulated mt-aaRSs in Group C exert additional effects on host cells upon SARS-CoV-2 infection.

In KEGG analysis, “aminoacyl-tRNA biosynthesis” was revealed as one of the enriched pathways overlapping three groups, reflecting canonical functions of aaRSs (Figure 5D). Nonetheless, the IN nodes from Groups A and C were mostly enriched in pathways of different viral infections including “coronavirus disease-COVID-19,” “human cytomegalovirus infection,” and “Kaposi sarcoma-associated herpesvirus infection,” suggesting a close link between mt-aaRSs and viral infections. In addition, the IN nodes from each group were also enriched in signaling pathways related to many types of cancers (Figure 5D). Taken together, the new sets of IN nodes fill the gap between the first interactors in response to SARS-CoV-2 and the downstream aaRSs, suggesting vital roles of a certain set of mt-aaRSs during SARS-CoV-2 infection.

## Discussion

To date, increasing evidence has proved the roles of some aaRSs in the immune regulation upon viral infections. For example, influenza A virus, a negative-sense single-stranded RNA virus, induces EPRS1 phosphorylation at S990, causing its release from the MSC. The released EPRS1 further protects mitochondrial anti-viral signaling protein (MAVS) from proteasomal degradation by poly(rC)-binding protein 2 (PCBP2) (Lee et al., 2016). AIMP1 is also upregulated in bronchial epithelial cells after influenza A infection, exhibiting a possible role for AIMP1 in response to viral infection (Lee et al., 2016). Nevertheless, the functional connection between aaRSs and SARS-CoV-2 infection remains unknown. The current mining-based symmetrically analysis revealed, for the first time, a strong correlation between human aaRSs and CoVs infections, especially the SARS-CoV-2 infection.

Clinical studies have revealed that 90% of COVID-19 patients manifest lymphocytopenia (Chan et al., 2020; Rothan and Byrareddy, 2020; Zhang et al., 2020), implying a unique inflammatory response induced by SARS-CoV-2. Elevated inflammatory factors were also found in COVID-19 patients with severe symptoms, thereby triggering a cytokine storm that induces disseminated damage to the host (Lang et al., 2020; Mehta et al., 2020; Pinney et al., 2020). In order to characterize the roles of aaRSs upon SARS-CoV-2 infection, we firstly summarized our analyses at mRNA levels. We found *aaRSs* are mostly downregulated in COVID-19 patient-derived tissues and SARS-CoV-2-infected cell lines (Figure 2). However, we are unclear about the roles of aaRSs based on their mRNA levels during SARS-CoV-2 infection, since differential expression can mean two things: the virus downregulates a host gene to benefit its replication and survival, or the host downregulates a gene to help combat the virus, and the same holds true for the upregulated genes. Therefore, further in-depth analyses and functional studies regarding the transcriptional changes of *aaRSs* are warranted.

To grasp the wider implications, we performed further analyses of aaRSs at different platforms, which provide vital information regarding their roles in COVID-19. It is interesting to note that most mt-aaRSs seem to play anti-viral roles based on their *Z* scores (Figure 1B). The discovery that CoV-proteins interact predominantly with mt-aaRSs, as well as the validation of the physical interaction between TARS2 and SARS-CoV-2 M protein did surprise us (Figures 4A,B). More strikingly, the enriched GO terms and KEGG pathways for the IN nodes from Group C reflected essential roles of the upregulated mt-aaRSs during SARS-CoV-2 infection (Figures 5A,D). In fact, numerous studies have revealed the association between viruses and mitochondria. During viral infections, mitochondria can be directly targeted by viral proteins (Thaker et al., 2019; Elesela and Lukacs, 2021). One theory is that virus-mitochondria interactions hamper MAVS (Refolo et al., 2020), a mitochondrial-localized anti-viral protein, leading to an inadequate host immune response. Indeed, one computational work suggested that the RNA of SARS-CoV-2 preferentially localizes to mitochondria (Wu K. E. et al., 2020). Another study based on global affinity purification mass spectrometry analysis further revealed putative interactions of SARS-CoV-2 proteins with human mitochondrial proteins (Gordon et al., 2020). A very recent work also confirmed that SARS-CoV-2 M protein directly interacts with MAVS and impairs MAVS-mediated innate anti-viral response (Fu et al., 2021). Hence, our findings suggested that mt-aaRSs are probably hijacked by SARS-CoV-2; the interaction between mt-aaRSs and SARS-CoV-2 proteins further alters energy production or intracellular stress, thus affecting host responses. In this regard, it is worthy of investigating more interactions between mt-aaRSs and SARS-CoV-2 proteins in the future.

Inspiringly, based on our analyses, TARS2, HARS2 and EARS2 stand out among all mt-aaRSs upon SARS-CoV-2 infection. TARS2 was uniformly downregulated at both mRNA and protein levels (Figures 2, 3A). The physical interaction between TARS2 and SARS-CoV-2 M protein further provided evidence for their close link (Figure 4B). In the contrary, HARS2 was significantly increased in COVID-19 patient-derived samples and the SARS-CoV-2-infected cell lines at protein levels (Figure 3A). The phosphorylated-HARS2 was also significantly increased at different time points after SARS-CoV-2 infection in Vero-E6 cells (Figure 3D). Although we did not find the physical interaction between HARS2 and SARS-CoV-2 M protein (Figure 4B), the enriched GO and KEGG analyses of the IN nodes from Group C, in which HARS2 was included, revealed multiple roles related to viral infections (Figures 5A,D), implying that HARS2 is a potential key factor in COVID-19. In addition, *EARS2* got significant negative *Z* scores across infections with three CoVs (Figure 1A), and it was also revealed as a significant IN gene overlapping three groups upon SARS-CoV-2 infection (Table 1). All these findings pinpointed that EARS2 is very likely to be involved in the process of CoVs infections, and especially serves as a critical factor mediating SARS-CoV2 infection in the host. Of interest, some aaRSs have been revealed to target mRNAs through binding tRNA-like elements, suggesting a mode of mRNA regulation by aaRSs (Levi and Arava, 2019; Garin et al., 2021). In addition, RNA viruses can hijack host cellular machinery by mimicking the tRNA-like structure to trick host aaRSs (Bonilla et al., 2021). Although there has been no study to report the tRNA-like elements regulated by TARS2, HARS2 or EARS2, the investigation of the aaRSs-mediated control of viruses or host mRNAs, as well as the function of tRNA-like structures will reveal novel molecular mechanisms for COVID-19.

Furthermore, it is worth noting that the IN nodes in each group were enriched in pathways linked to multiple cancers (Figure 5D). Since angiogenesis has been regarded as one of the hallmarks of cancer (Viallard and Larrivee, 2017), the cancer-related pathways may be an indication of vascular remodeling. Indeed, a recent work reported that the lung tissues of COVID-19 patients present significant angiogenesis (Ackermann et al., 2020). Interestingly, EGFR, an overlapped IN node (Table 1), is also a vital intermediate in angiogenesis (Sharma et al., 2021). Thus, these IN nodes enriched in cancer-related pathways may suggest non-canonical functions (e.g., vascular remodeling) of aaRSs involved in COVID-19.

**FIGURE 6:**
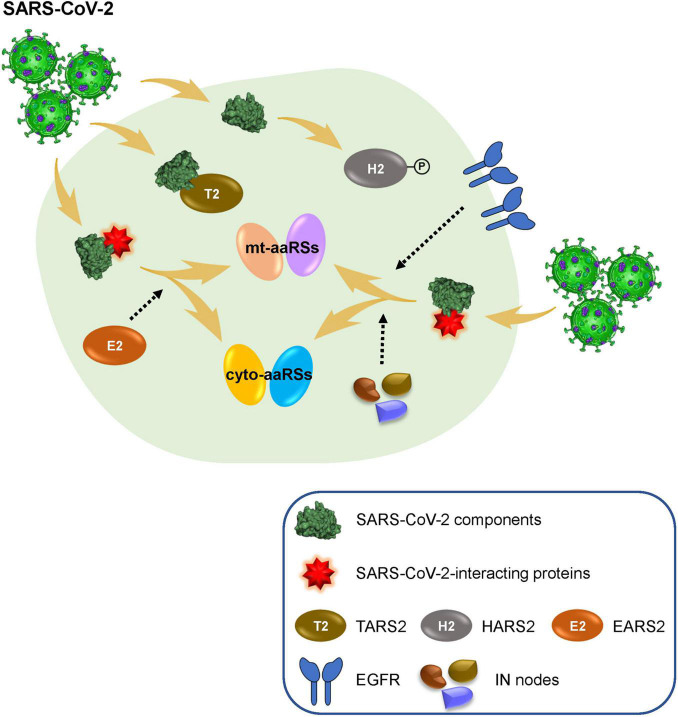
Human aminoacyl-tRNA synthetases (aaRSs) involved in SARS-CoV-2 infection. SARS-CoV-2 infection leads to obvious changes of human mt-aaRSs, in which, TARS2 physically interacts with SARS-CoV-2 M protein; the phosphorylation of HARS2 is significantly increased; and EARS2 serves as a key intermediate (IN) node mediating SARS-CoV-2 infection in the host. In addition, newly identified IN nodes such as EGFR fill the gap between the first interactors in response to SARS-CoV-2 and the downstream aaRSs, reflecting vital roles of certain aaRSs during SARS-CoV-2 infection.

In sum, we collected available omics data to investigate correlation between CoVs and human aaRSs, with a particular focus on their connections during SARS-CoV-2 infection. We found a strong involvement of mt-aaRSs in response to SARS-CoV-2 infection, and identified TARS2, HARS2, and EARS2 as potential key factors during this process (Figure 6). This work inspires future efforts to thoroughly understand the function of aaRSs during CoVs infections. More importantly, our analyses may lead to new therapeutic strategies in human infections by targeting certain aaRSs. Aside from being used as an anti-viral agent, aaRSs could also be developed as adjuvants for vaccination. This possibility, together with the aforementioned opportunities for therapies, needs thorough mechanistical investigations on aaRSs in the future.

## Data Availability Statement

The original contributions presented in the study are included in the article/Supplementary Material, further inquiries can be directed to the corresponding authors.

## Author Contributions

YF and KT collected the data and performed the analysis. QL, JL, and MF assisted the analysis. Z-WZ, HC, and XD assisted the design of the project. HZ and LS designed the project and wrote the manuscript. All authors approved the final manuscript.

## Conflict of Interest

The authors declare that the research was conducted in the absence of any commercial or financial relationships that could be construed as a potential conflict of interest.

## Publisher’s Note

All claims expressed in this article are solely those of the authors and do not necessarily represent those of their affiliated organizations, or those of the publisher, the editors and the reviewers. Any product that may be evaluated in this article, or claim that may be made by its manufacturer, is not guaranteed or endorsed by the publisher.

## Supplementary Material

Supplementary Figure 1 Interaction network between aminoacyl-tRNA synthetases (aaRSs) and different viruses. Green and red circles represent aaRSs and viruses, respectively. The size of the circle represents the complexity of the interaction. The larger circle represents the more complex interaction network.

Supplementary Figure 2 *Z* scores of cytoplasmic (cyto) and mitochondrial translation- and ribosome-related genes in Vero-E6 cells challenged with different coronaviruses (CoVs). Transcriptional expressions of translation- and ribosome-related genes (blue), translation-related genes (green), and ribosome-related genes (red) based on a genome-wide CRISPR screening were clustered by their mean *Z* scores with red representing *Z* > 0 while blue representing *Z* < 0. **p* < 0.05; ***p* < 0.01 (vs. mock-infected cells).

Supplementary Figure 3 Transcriptional profiles of cytoplasmic (cyto) and mitochondrial translation- and ribosome-related DEGs in patient-derived samples and cell lines infected with SARS-CoV-2. Heatmaps of translation- and ribosome-related DEGs (with the cyto DEGs on the left and the mitochondrial DEGs on the right) in BALF, post-mortem lung tissues from coronavirus disease 2019 (COVID-19) patients, as well as in NHBE and A549-ACE2 cells infected with SARS-CoV-2. The fold change in gene expression is indicated by color intensity, with red representing the upregulated DEGs and blue representing the downregulated DEGs. The DEGs include translation- and ribosome-related genes (blue), translation-related genes (green), and ribosome-related genes (red).

Supplementary Figure 4 Cytoplasmic (cyto) translation- and ribosome-related DEPs and the total protein levels of certain aminoacyl-tRNA synthetases (aaRSs). **(A)** Heatmap of cyto translation- and ribosome-related DEPs from different samples. The fold change in protein expression is indicated by color intensity, with red representing the upregulated DEPs and blue representing the downregulated DEPs. The DEPs include translation- and ribosome-related proteins (blue), and translation-related proteins (green). **(B)** The total protein levels of CARS1, DARS1 and HARS2. Ctrl, control; Inf, infection; hr, hour; ns, not significant (vs. mock-infected cells at 0 h).

Supplementary Table 1 Interactions between human aminoacyl-tRNA synthetases (aaRSs) and viruses.

Supplementary Table 2 Primers used for cloning.

Supplementary Table 3 Transcriptional and protein profiles of aminoacyl-tRNA synthetases (aaRSs) in patient-derived samples and SARS-CoV-2-infected cell lines.

Supplementary Table 4 Groups of differential aminoacyl-tRNA synthetases (aaRSs) proteins.

Supplementary Table 5 Significant IN nodes and their enriched GO terms and KEGG pathways in each group.
