# Supplementary material for: The Landscape of Aminoacyl-tRNA Synthetases Involved in Severe Acute Respiratory Syndrome Coronavirus 2 Infection
Source: Front Physiol. 2022 Jan 26;12:818297. doi: 10.3389/fphys.2021.818297 (PMC8826553; doi:10.3389/fphys.2021.818297)

# Supplementary Figure 1

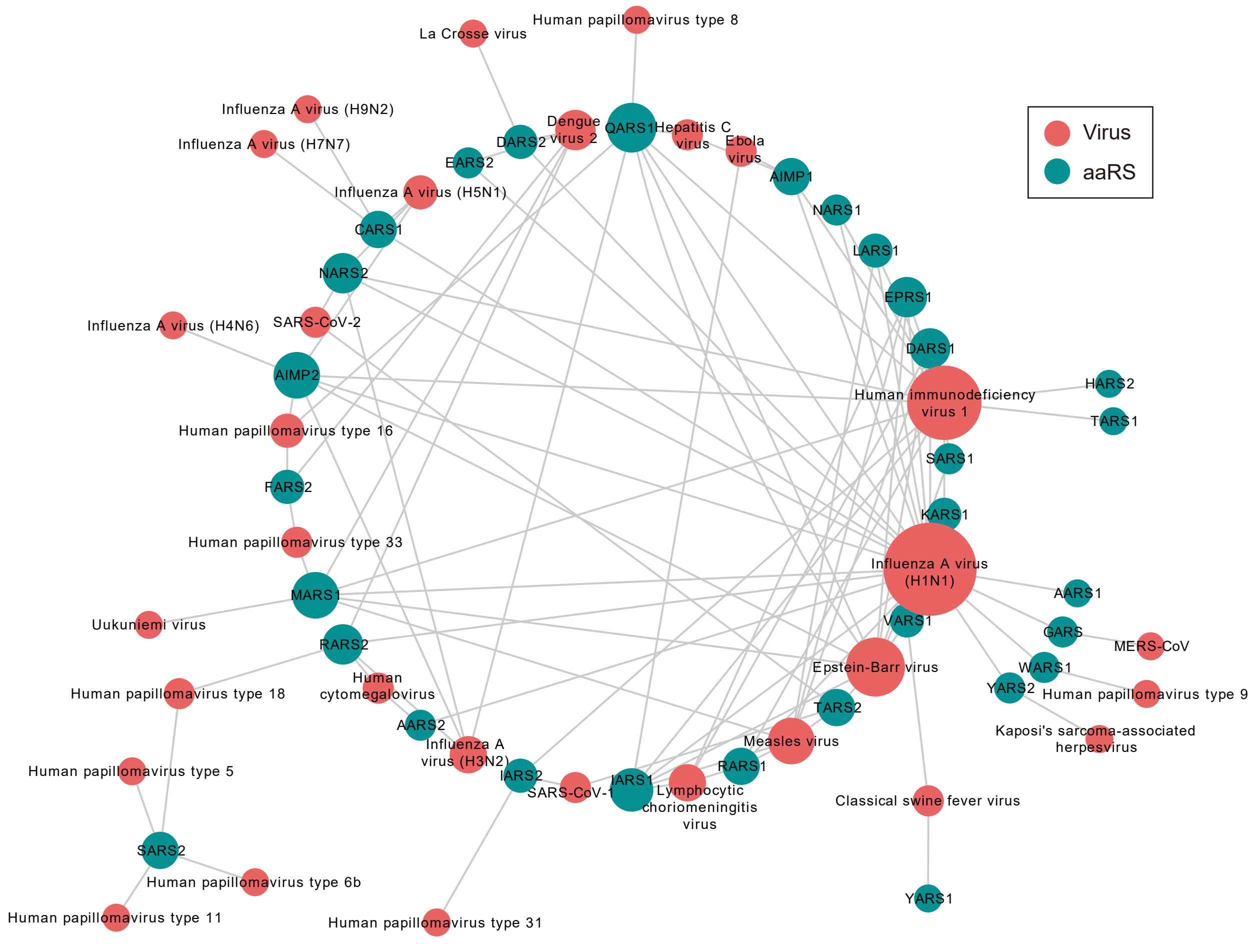

# Supplementary Figure 2

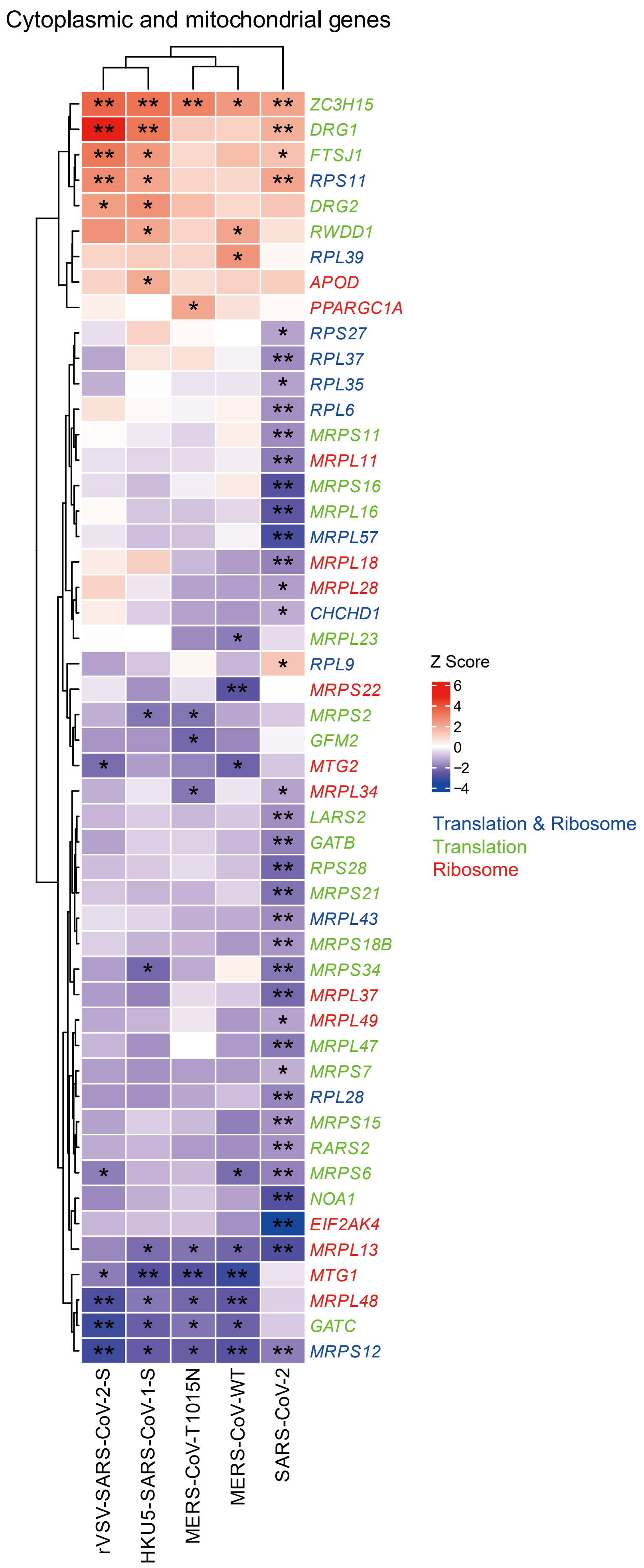

# Supplementary Figure 3

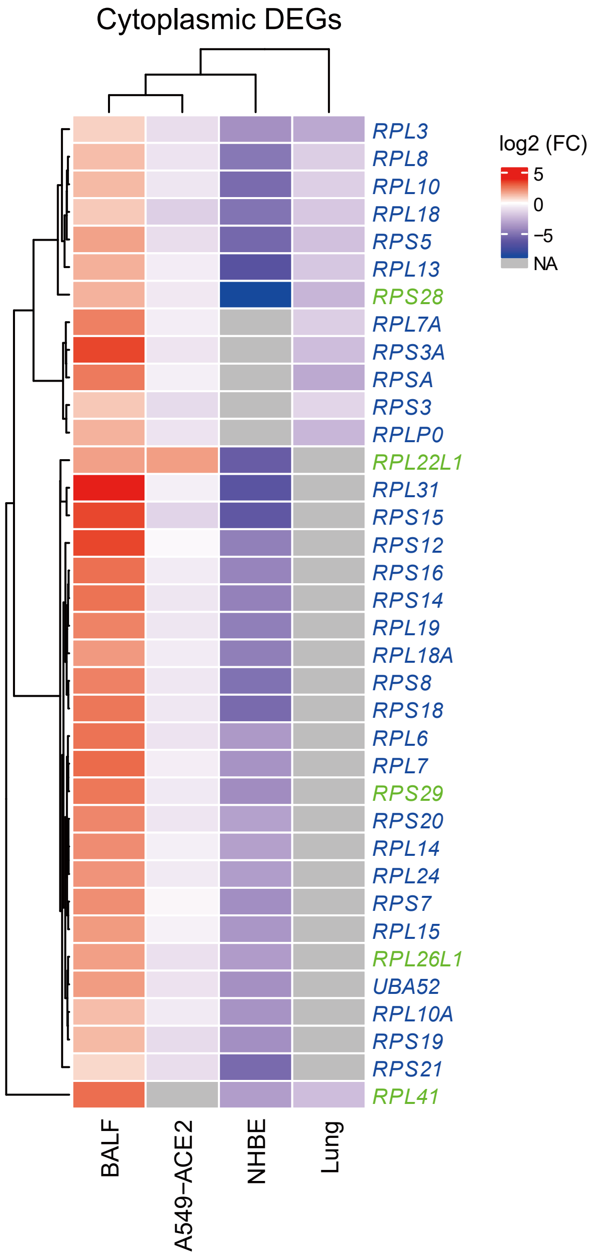


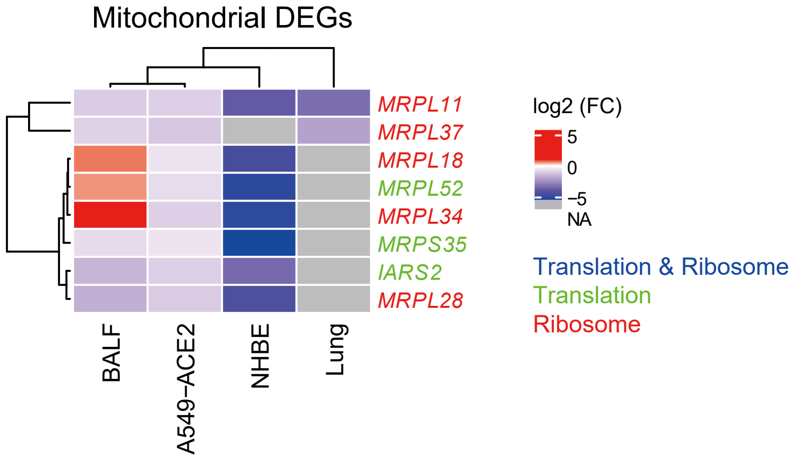

## Supplementary Figure 4

A

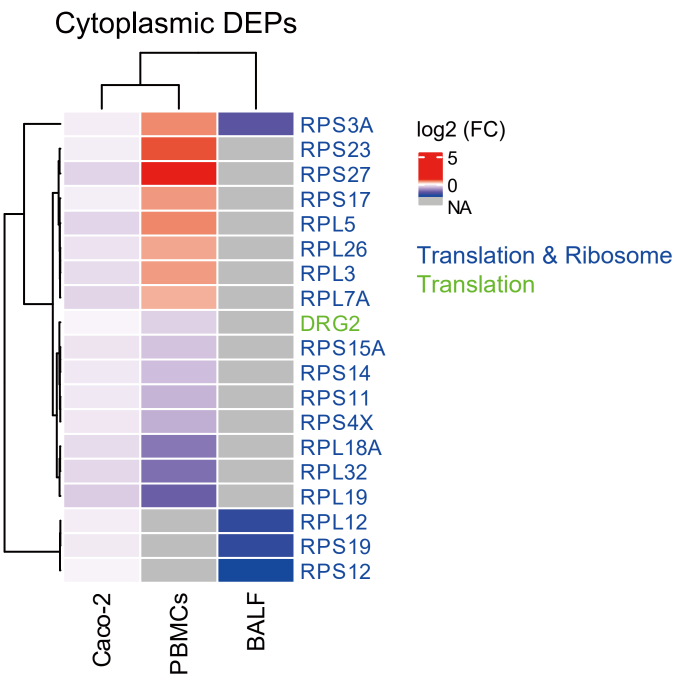


B

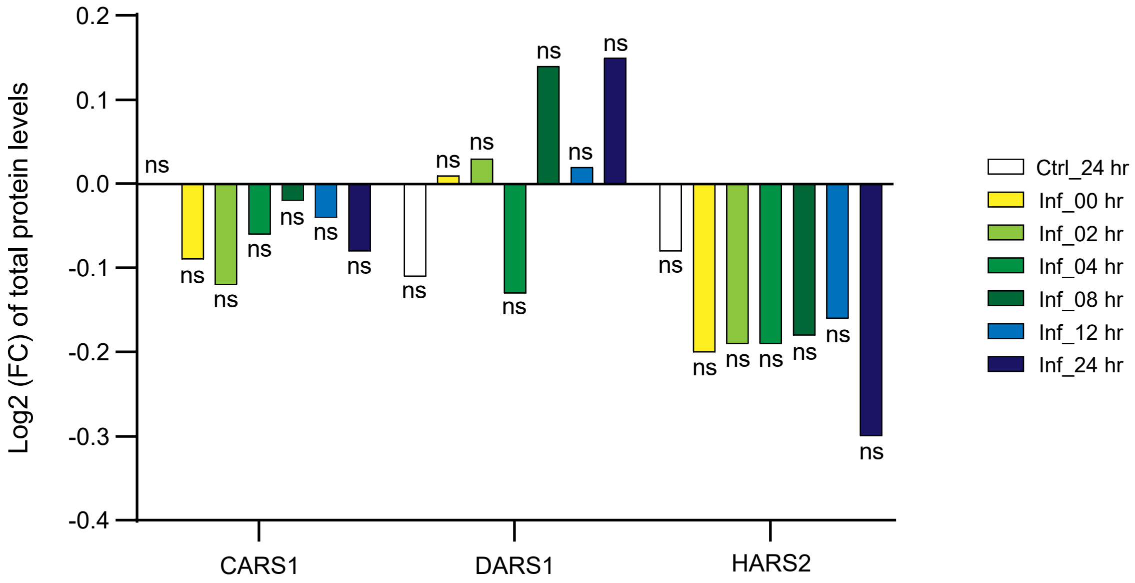

Supplement: Supplementary Figure 1 — Interaction network between aminoacyl-tRNA synthetases (aaRSs) and different viruses. Green and red circles represent aaRSs and viruses, respectively. The size of the circle represents the complexity of the interaction. The larger circle represents the more complex interaction network. [file Data_Sheet_1.PDF]
